# A Method for Industrial Smoke Video Semantic Segmentation Using DeffNet with Inter-Frame Adaptive Variable Step Size Based on Fuzzy Control

**DOI:** 10.3390/s26061949

**Published:** 2026-03-20

**Authors:** Jiantao Yang, Hui Liu

**Affiliations:** Faculty of Information Engineering and Automation, Kunming University of Science and Technology, Kunming 650500, China; yangjiantao226@126.com

**Keywords:** smoke segmentation, LR-ASPP, non-rigid deformation, adaptivity, fuzzy control, DeffNet

## Abstract

Segmenting non-rigid objects such as smoke in video requires effective utilization of temporal information, which remains challenging due to their irregular deformation and complex appearance variations. Based on our previously proposed DeffNet for industrial fumes video segmentation, this letter presents a novel adaptive frame selection algorithm that employs fuzzy logic control to dynamically optimize the temporal processing step size for the specific task of industrial smoke video segmentation. Our method quantifies inter-frame variation using the Structural Similarity Index (SSIM) and Normalized Cross-Correlation (NCC) as inputs to a fuzzy inference system. Gaussian membership functions, shaped via K-means clustering, and a five-rule fuzzy system are designed to determine the optimal step size, maximizing informative dynamic feature extraction while minimizing redundant computation. As a lightweight front-end module, the algorithm integrates seamlessly into the existing DeffNet segmentation framework without reconstructing new network architecture. Extensive experiments on a dedicated industrial smoke video dataset demonstrate that our approach effectively improves the segmentation performance of DeffNet, achieving 84.27% Intersection over Union (IoU) while maintaining a high inference speed of 39.71 FPS. This work provides an efficient and scene-specific solution for temporal modeling in industrial smoke non-rigid object segmentation and offers a practical improved strategy for DeffNet in real-time industrial smoke monitoring.

## 1. Introduction

With the rapid advancement of modern industrial technology, industrial soot emissions have continued to rise. Such soot particles not only directly degrade the atmospheric environment but also pose a threat to human health through air propagation [1,2,3]. According to statistics from the World Health Organization (WHO), millions of deaths annually are attributed to respiratory diseases, cardiovascular disorders, and other health issues associated with soot pollution [3].

In the monitoring of industrial soot emissions, soot darkness density serves as a core indicator. The mainstream determination method relies on the Ringelmann scale observation, where pollution levels are identified by manually comparing soot plumes with the Ringelmann chart. However, this approach is characterized by inefficiency (i.e., time-consuming and labor-intensive) and low accuracy due to human subjectivity. Meanwhile, sensor-based detection methods face challenges such as high implementation costs and cumbersome subsequent maintenance.

Traditional manual feature-based image segmentation methods are constrained by the performance of computer CPUs, enabling the extraction of only low-level features (e.g., color, spatial structure, and texture) for segmentation tasks. Representative techniques in this category include threshold segmentation [4,5], texture analysis [6,7], inter-frame difference [8,9], and region-based segmentation [10,11].

**Figure 1:**
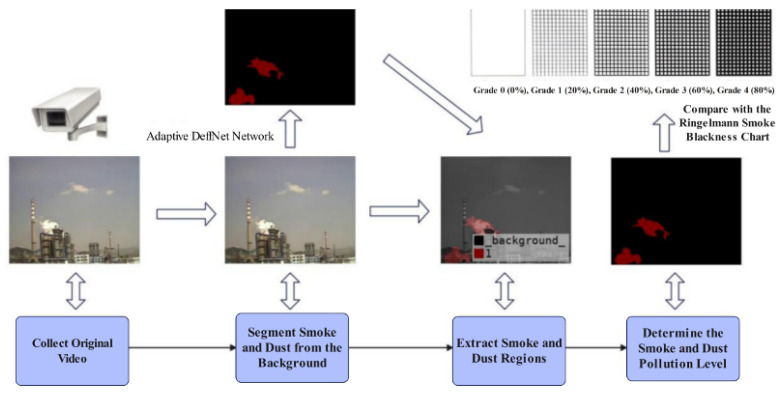
Schematic diagram of industrial smoke dust division.

In recent years, computer vision technology [12,13] has undergone rapid development and been widely applied in fields such as semantic segmentation and object detection, providing a novel approach for industrial soot pollution level monitoring. Specifically, through computer-aided processing and analysis of images or videos, rapid and accurate assessment of soot pollution grades can be achieved. Among various techniques, video segmentation [14,15] plays a crucial role: it first separates the soot region from the background, and then the extracted soot region is compared with the Ringelmann smoke darkness chart to complete the pollution level determination. Focusing on the application of computer vision in industrial soot pollution monitoring [16,17], this study addresses four key phases: (1) acquisition of raw soot data; (2) precise segmentation of soot regions from the background using computer vision algorithms; (3) extraction of target soot information; and (4) Accurately determine the pollution level by referring to the Ringelman concentration [18,19] table (as shown in Figure 1). This research framework and technical approach are also applicable to key scenarios such as fire accident monitoring and early warning. The smoke produced during a fire accident is also a non-rigid deformation target, with irregular shapes and complex appearance changes similar to industrial smoke dust. The frame-based adaptive step size DeffNet method based on fuzzy decision proposed in this paper can achieve precise segmentation of non-rigid targets through efficient time modeling. It not only solves the technical pain points of industrial smoke dust monitoring but also provides a generalized technical solution for smoke recognition and monitoring in fire accidents.

In the domain of smoke visual monitoring algorithms, researchers have leveraged diverse models, including Back Propagation (BP) neural networks, transfer learning integrated with Fully Convolutional Networks (FCNs), dual-path networks, and multi-scale FCNs, to carry out investigations into smoke target detection, pixel-level segmentation of smoke across multi-scenario settings, and related tasks, yielding a series of progressive achievements [20,21,22,23]. Nevertheless, these existing approaches are predominantly direct applications or routine modifications of general-purpose visual task models, which lack tailored design that fully incorporates the distinct characteristics of smoke-related datasets and thus cannot achieve optimal adaptation to the specific data attributes of smoke targets.

On the one hand, convolutional neural networks (CNNs) inherently possess the capability to learn spatial features but lack the ability to capture temporal features. Consequently, some researchers have evolved image semantic segmentation into video semantic segmentation. For instance, Benoughidene, A. et al. [24] proposed the FCN-LSTM model. Nevertheless, due to the high similarity between consecutive video frames, the convLSTM [25] component failed to learn substantial discriminative features, leading to limited improvement in model accuracy and failure to meet real-time requirements. On the other hand, Other scholars have explored the integration of optical flow for auxiliary enhancement: for example, Gadde et al. [26] developed NetWarp-PSPNet, which incorporates optical flow into existing image semantic segmentation models. However, DIS Flow (the optical flow method adopted) is sensitive to changes in light intensity, making it difficult to track the movement of industrial exhaust gases. In contrast, the DeffNet proposed by our research team effectively extracts soot motion information by leveraging the non-rigid motion characteristics of soot, thereby enabling effective differentiation from the background and achieving better segmentation performance in industrial smoke video segmentation. However, DeffNet adopts a fixed two-frame temporal processing strategy, which cannot adapt to the dynamic variation of industrial smoke in complex scenes, leading to redundant computation or insufficient extraction of dynamic features—this is the key limitation we aim to address in this study.

Building on the authors’ previous work on DeffNet [27]—which demonstrated superior performance in extracting dynamic features for video semantic segmentation and achieving finer segmentation results—this study aims to conduct targeted step-by-step optimization on DeffNet to segment complete and accurate soot targets. Maximizing the extraction of soot dynamic features constitutes the core focus of this research. To this end, an inter-frame adaptive DeffNet based on fuzzy decision-making is proposed.

A large number of validation experiments conducted on a challenging self-built industrial smoke video dataset have shown that the method proposed in this paper achieves the current optimal performance in the industrial smoke segmentation task. At the same time, comparative experiments with the original DeffNet [27], NetWarp and other mainstream static/dynamic segmentation models indicate that the proposed adaptive frame selection method based on the fuzzy controller significantly improves the segmentation accuracy (IoU) while ensuring the real-time inference speed.

1. We propose a lightweight, plug-and-play fuzzy temporal adapter that intelligently regulates inter-frame sampling rates, achieving a superior efficiency-accuracy trade-off for the specific task of industrial smoke non-rigid object segmentation. This adapter is integrated into the existing DeffNet framework without changing its original network structure.
2. We present a data-centric methodology for automatically constructing the fuzzy system, eliminating the need for manual hyperparameter tuning and enhancing generalizability.
3. Extensive validation on a challenging industrial smoke dataset confirms that our method effectively improves the segmentation accuracy and scene adaptability of DeffNet, boosting segmentation accuracy (IoU) while operating at real-time speeds in industrial smoke monitoring scenarios.

## 2. Industrial Smoke Segmentation Method Based on Inter-Frame Adaptive DeffNet with Fuzzy Decision-Making

### 2.1. DeffNet Network Architecture

Firstly, the model takes two consecutive RGB frames and grayscale frames as input, totaling 8 channels (grayscale frames are used here to enable the temporal feature model to better learn deformation information). The input data is divided into two branches: the grayscale frames are fed into the mU-net [28] of the temporal feature model to predict the deformation field. After processing by STN (Spatial Transformer Network) [29], a sampling grid is obtained. The weights of this network are pre-trained. Once the sampling grid is generated, it is mapped back to the feature layer of the previous frame. Through bilinear interpolation warping, feature points are propagated via the sampling grid to generate a new feature layer, which has 16 channels.

**Figure 2:**
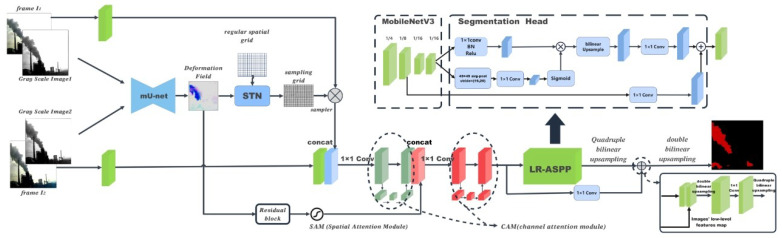
Network Structure Diagram of DeffNet.

This new feature layer is stacked with the feature layer of the current frame and then weighted by channel attention, resulting in a feature layer fused with dynamic information. On the other hand, the aforementioned deformation field is passed through residual blocks and a spatial attention module in sequence to generate a 16-channel feature map. This feature map is stacked with the previously obtained dynamic information-fused feature layer, followed by another fusion via the channel attention module. Finally, the fused feature layer undergoes one convolution operation first, then dimension reduction via 1×1 convolution, and is subsequently introduced into the decoder layer for an element-wise addition operation with the decoder layer features. After processing by the multi-resolution LR-ASPP (Lite Atrous Spatial Pyramid Pooling) module, smoke and background segmentation is achieved. The overall network architecture of DeffNet is shown in Figure 2.

This chapter mainly introduces the core structure and working principle of the inter-frame adaptive DeffNet framework. The core innovation of this framework is to integrate the fuzzy logic controller used for adaptive frame selection into the original DeffNet network [27]. To achieve more refined and efficient industrial smoke segmentation, the framework proposed in this study introduces fuzzy decision-making on the basis of the DeffNet network previously proposed by the author [27], to adaptively determine the optimal number of stacked frames for extracting the deformation field. Although the original DeffNet network can effectively extract the dynamic deformation information of industrial smoke, it uses a fixed 2-frame inter-frame sampling step, which results in redundant calculations for slow-changing smoke and fails to fully capture the dynamic characteristics of fast-changing smoke. Therefore, this study, based on the DeffNet network, first increases the total amount of smoke deformation information that can be extracted by stacking the deformation fields between different frames and then adaptively determines the optimal number of stacked frames according to the real-time change speed of the smoke using the fuzzy logic controller.

### 2.2. Theory of Inter-Frame Adaptive DeffNet

The fuzzy inference system takes the original SSIM and NCC values of consecutive frames as input variables without additional normalization processing, and the quantitative mapping of input features is realized by the subsequent K-means clustering algorithm. (All the design and optimization of the fuzzy inference system are based on 10 industrial smoke videos, with the first 100 frames selected for each video). Gaussian membership functions for fuzzification are constructed in a data-driven manner: K-means clustering is performed on the SSIM and NCC values of the experimental dataset first, the obtained cluster centers are used as the mean parameters of Gaussian membership functions, and the intra-cluster variances are taken as the variance parameters. On this basis, the membership function of the output step size is further constructed by fusing the membership degrees of SSIM and NCC. The fuzzy rules are designed according to the smoke change speed, and the centroid method is used for defuzzification to determine the specific optimal frame number, and the optimal step size is output accordingly for frame selection.

**Figure 3:**
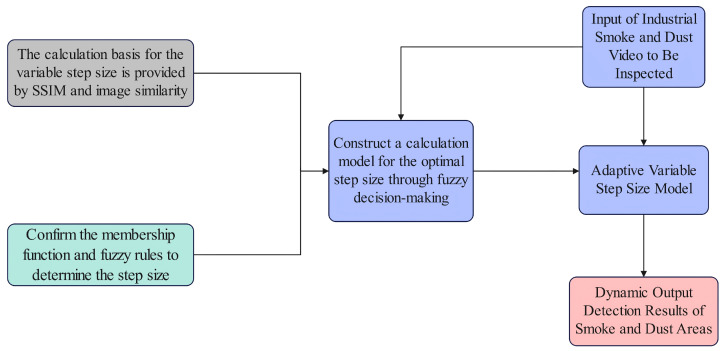
Flowchart of Industrial Smoke Segmentation Method Using Inter-Frame Adaptive DeffNet Based on Fuzzy Decision-Making.

This section mainly introduces the network-related structures. To achieve more refined network segmentation, the network proposed in this study incorporates fuzzy decision-making to determine the optimal number of frames, based on the DeffNet network previously proposed by the author [30]. Although the DeffNet network can effectively extract dynamic information about smoke, it uses a fixed number of two frames. Therefore, building on the DeffNet network, this study increases the amount of smoke deformation information by stacking deformation fields between different frames, and then applies fuzzy decision-making to determine the optimal number of stacked frames. The detailed description is as follows:

First, two indicators are introduced to judge the speed of smoke changes: the first is the Structural Similarity Index (SSIM), and the second is image similarity. The speed of industrial smoke changes is evaluated using these two-dimensional indicators, and the optimal number of frames is determined based on this evaluation. The framework diagram of this article is shown in Figure 3.

#### 2.2.1. Adaptive Step-Size Mechanism for Industrial Smoke Segmentation Based on Fuzzy Decision-Making

Fuzzy decision-making theory is a control method based on fuzzy logic, suitable for systems where accurate mathematical models are difficult to establish or model uncertainties exist. For the uncertainty, variability, and complexity of current smoke video data, fuzzy decision-making exhibits strong adaptability, enabling it to address control issues in nonlinear systems and fuzzy environments. Its expression form, based on expert experience and rules, is intuitive and easy to understand, interpret, and adjust. Additionally, this method can effectively handle system fuzziness and uncertainty, enhancing the network’s adaptability to changes in industrial smoke environments. Thus, fuzzy decision-making theory demonstrates excellent adaptability and robustness when processing smoke data.

The fuzzy inference system proposed in this paper is a hybrid method combining expert prior knowledge and data-driven optimization, which does not completely eliminate manual intervention but significantly reduces the workload of manual hyperparameter tuning. Specifically, a set of initial fuzzy rules are first formulated based on the expert experience in industrial smoke monitoring, and the rationality and effectiveness of these initial rules are fully verified by the quantitative experimental results in Figures 5 and 6. On this basis, the K-means clustering algorithm is introduced to realize the data-driven construction of the Gaussian membership function, which further optimizes the performance of the fuzzy inference system without additional manual adjustment of membership function parameters.

When dealing with different smoke change speeds, it is necessary to select appropriate reference step sizes and fuzzy rules based on the speed of smoke changes, and the core rule is uniformly defined as: “For slow smoke changes, a larger step size is adopted; for fast smoke changes, a smaller step size is adopted”. Specific implementation principles include:

(1) **Adapting to slow change speed**: For slow smoke changes, a larger step size is adopted to avoid redundant computation while capturing the subtle deformation features of smoke, and a set of simplified fuzzy rule matching strategies is applied to maintain the efficiency of feature extraction.
(2) **Avoiding information loss in fast changes**: An excessively large step size for fast-changing smoke will cause the loss of key dynamic deformation information due to significant inter-frame variations, leading to segmentation inaccuracy. Thus, a smaller step size is adopted to track the rapid deformation of smoke in real time.
(3) **Capturing rapid deformation features**: For fast smoke changes, the smaller step size can ensure that the system captures every key frame of smoke deformation, avoiding the omission of dynamic information and improving the accuracy of edge and dynamic region segmentation.
(4) **Balancing efficiency and accuracy**: The core rule of “slow change → larger step size, fast change → smaller step size” is designed to balance the efficiency and accuracy of industrial smoke segmentation—larger step sizes reduce redundant computation for slow changes, while smaller step sizes ensure feature integrity for fast changes, both meeting the real-time requirements of industrial on-site monitoring.

**Figure 4:**
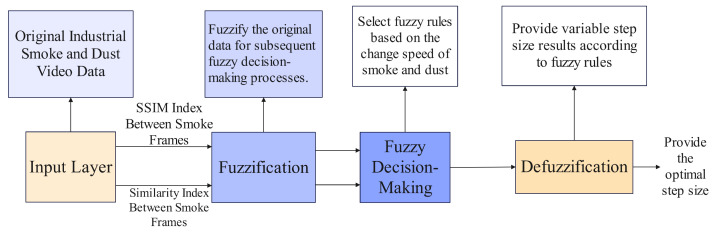
Principle of Adaptive Step Size for Industrial Smoke Segmentation Based on Fuzzy Decision-Making.

The core of fuzzy decision-making is the fuzzy controller, whose workflow is shown in Figure 4.

**Figure 5:**
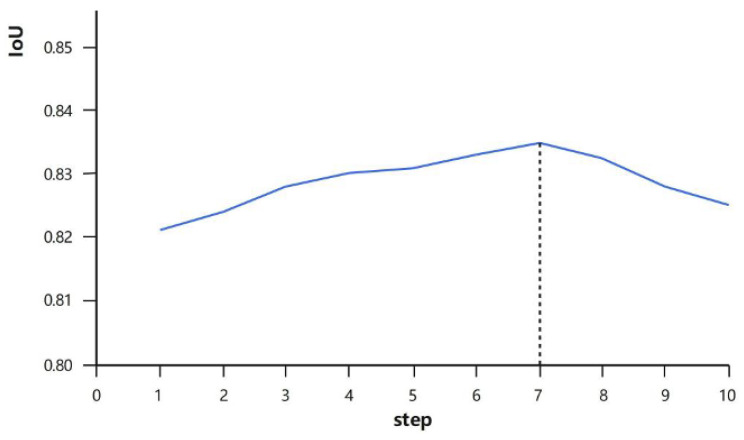
Diagram of the Relationship Between Optimal Step Size and Accuracy.

To verify the conjecture proposed in this study, data from three videos were selected for feasibility validation. First, an experiment on the optimal step size was conducted: the videos were first frame-segmented, and new frames were continuously stacked onto each frame to observe whether an optimal step size exists. Here, IoU (Intersection over Union) represents the accuracy, and “step” denotes the optimal step size. The relationship between the optimal step size and accuracy is shown in Figure 5.

It can be observed that as the step size continues to stack, there exists an optimal step size: when segmenting the current frame of the video, the 7th frame achieves the highest accuracy and thus serves as the optimal step size. After the 7th frame, the accuracy gradually decreases. This study analyzes that the reason may be that the difference between subsequent frames and the first frame becomes increasingly significant, which leads to errors in the extracted dynamic information and consequently results in a decline in accuracy.

**Figure 6:**
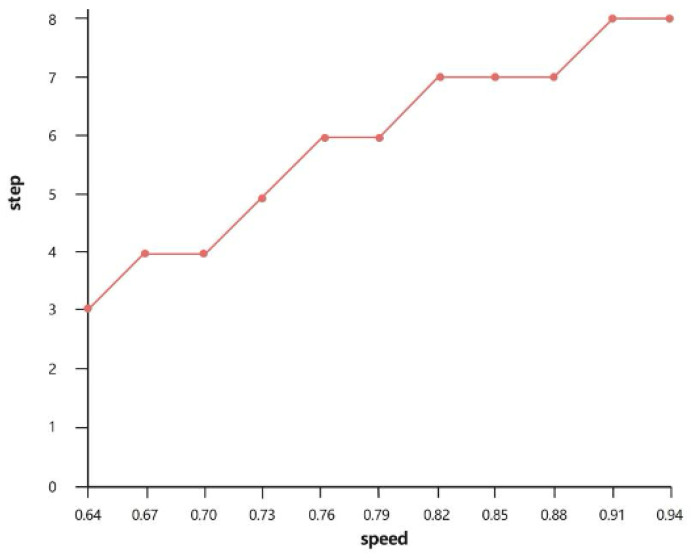
Diagram of the Relationship Between Smoke Change Speed and Optimal Step Size.

To verify the relationship between the smoke change speed and the optimal step size, this study correlates the smoke change status of the segmented current frame with the optimal step size under the video data. Here, “Speed” represents the average value of the sum of SSIM (Structural Similarity Index) and image similarity between the current frame and the frame corresponding to the optimal step size, as shown in Figure 6 below.

Therefore, this study clearly defines and unifies the fuzzy rule between the smoke change speed and the optimal step size: a slower smoke change → a larger step size, and a faster smoke change → a smaller step size. This rule is consistently applied throughout the entire study, and the fuzzy decision-making system is constructed based on this core logic to determine the optimal step size for each frame of industrial smoke video.

The rationality and effectiveness of the designed fuzzy rules have been fully verified through quantitative experimental results, as shown in Figure 5 and Figure 6. All experiments were based on 10 industrial smoke videos (the first 100 frames of each video were selected). Figure 6 (the relationship between the speed of smoke change and the optimal step size) is the core experimental basis. It quantified the one-to-one correspondence between the speed of smoke change (represented by SSIM/NCC values) and the optimal step size, and directly proved the validity of the fuzzy rule “a large step size is suitable for situations with slow smoke change, and a small step size is suitable for situations with rapid smoke change”. Figure 5 (the relationship between the optimal step size and the segmentation accuracy) serves as supplementary verification, quantifying the actual segmentation performance corresponding to the optimal step size output by the fuzzy rules and further confirming the rationality of the rule design.

#### 2.2.2. Indicators for Measuring Smoke Change Rate

The rationality and effectiveness of the initial fuzzy rules formulated based on expert experience have been fully verified by the quantitative experiments in Figure 5 and Figure 6, but the manual setting of Gaussian membership function parameters still has the problems of strong subjectivity and limited adaptability to industrial smoke scene characteristics. To solve this issue and further reduce manual intervention, the K-means clustering algorithm is introduced to realize the data-driven construction of membership function parameters. From the SSIM and NCC values of the experimental data, the clustering centers are automatically learned, and these clustering centers are directly used as the mean parameters of the Gaussian membership function, while the intra-class variance is used as the variance parameter. This data-driven method for constructing membership function parameters based on K-means clustering not only further reduces manual intervention and subjective bias caused by manual parameter setting, but also makes the membership function more consistent with the data characteristics of industrial smoke videos, thus improving the adaptability and generalization ability of the fuzzy system. On the basis of the expert rules verified by quantitative experiments, this method further optimizes the performance of the fuzzy reasoning system and ensures the accuracy of step size decision-making for industrial smoke segmentation.

Two indicators are used herein to measure the smoke change rate: the first is SSIM, and the second is the Normalized Cross-Correlation (NCC, i.e., image similarity). SSIM, short for Structural Similarity Index, is an indicator for comparing and evaluating image similarity. It takes into account not only the brightness and contrast of images but also their structural information, thus enabling a more comprehensive description of the similarity between two images.

The Structural Similarity Index (SSIM) is commonly applied in the field of image processing to quantify the similarity between two images. It considers three aspects: brightness, contrast, and structure, and calculates the similarity between two images by comparing their similarity in these aspects. The value of SSIM ranges from (−1,1): a value closer to 1 indicates higher similarity between the two images, while a value closer to −1 indicates greater differences between them.

The calculation method of SSIM involves statistical variables such as the mean, variance, and covariance of images and simultaneously considers the weights of the three aspects (brightness, contrast, and structure). By calculating these statistical variables and weights, SSIM can provide a more comprehensive and accurate measure for describing image similarity, which is of great significance for applications such as image quality assessment and image compression.

The average gray level of an image is used as an estimate for brightness measurement.

(1) $$u_{x}=\frac{1}{H\times M}\sum_{i=1}^{H}\sum_{j=1}^{M}X\left(i,j\right)$$

The brightness comparison function for the two images

(2) $$l\left(x,y\right)=\frac{2u_{x}u_{y}+C_{1}}{u_{x}^{2}+u_{y}^{2}+C_{1}}$$

##### Contrast Comparison Function

The standard deviation of an image is used as the estimate for contrast measurement.

(3) $$\sigma x=\frac{1}{H\times W-1}\sum_{i=1}^{H}\sum_{j=1}^{W}{\left({\left(X\left(i,j\right)-u_{x}\right)}^{2}\right)}^{\frac{1}{2}}$$

Thus, the contrast comparison function for the two images

(4) $$c\left(x,y\right)=\frac{2\sigma_{x}\sigma_{y}+C_{2}}{\sigma_{x}^{2}+\sigma_{y}^{2}+C_{2}}$$

##### Structural Comparison Function


(5) $$s\left(x,y\right)=\frac{\sigma_{xy}+C_{3}}{\sigma_{x}\sigma_{y}+C_{3}}$$

##### SSIM Measurement Function

Finally, the above three comparison functions constitute the SSIM function.

(6) $$\mathrm{SSIM}\left(x,y\right)=\frac{\left(2u_{x}u_{y}+C_{1}\right)\left(2\sigma_{x}\sigma_{y}+C_{2}\right)}{\left(u_{x}^{2}+u_{y}^{2}+C_{1}\right)\left(u_{x}^{2}+u_{y}^{2}+C_{1}\right)}$$

where $u_{x}$ denotes the mean value and σ denotes the variance; *C* is a constant.

The second indicator is the image similarity index, which adopts the Normalized Cross-Correlation (NCC). NCC is a method for measuring the correlation degree between targets to be matched. During the matching process, first, an arbitrary pixel (px,py) in the original image is selected to construct a 3×3 neighborhood as the matching window. Then, a 3×3 matching window is also constructed for the target pixel position (px + d,py), followed by similarity measurement. Before calculating NCC, the images need to be processed to ensure horizontal position correction of the two frames, i.e., the optical centers are aligned on the same horizontal line—this ensures that the epipolar lines remain horizontal. Without such correction, the matching process can only be performed along the direction of inclined epipolar lines, which consumes more computing resources. Therefore, image correction is a crucial step to ensure the accuracy and efficiency of matching.

The calculation formula of NCC is as follows:

(7) $$NCC\left(I_{1},I_{2}\right)=\frac{\sum_{\left(x,y\right)\in w^{p}}\left(I_{1}\left(x,y\right)-u_{1}\right)\left(I_{2}\left(x+d,y\right)-u_{2}\right)}{\sqrt{\sum_{\left(x,y\right)\in w^{p}}{\left(I_{1}\left(x,y\right)-u_{1}\right)}^{2}\sum_{\left(x,y\right)\in w^{p}}{\left(I_{2}\left(x+d,y\right)-u_{2}\right)}^{2}}}$$

$I_{1}\left(x,y\right)$ represents the pixel value of the original image, and $u_{1}$ denotes the mean value of pixels within the original window. $I_{2}\left(x+d,y\right)$ stands for the pixel value of the original image at the position corresponding to the target image after the x-direction offset of *d*, while $u_{2}$ is the mean value of pixels in the matching window of the target image. $w^{p}$ refers to the aforementioned matching window, which is set to a size of 3×3. The matching window is moved across the target image from left to right and top to bottom. After each one-pixel movement, the pixel values within the current window and the template window are calculated, and the result is compared with a threshold value—if the result exceeds the threshold, the position is recorded.

If the NCC value is close to 1, it indicates that the image information of the two matching windows has a high correlation; conversely, if the NCC value is close to −1, it means the image information of the two matching windows is completely uncorrelated.

In this study, the range of selected frames is determined by quantizing the membership degrees (obtained from the two indicators of input structural similarity and image similarity) into specific frames. The output range of frames is defined as between 1 and 10.

#### 2.2.3. Validation of SSIM and NCC for Characterizing Smoke Dynamic Characteristics

To verify that SSIM and NCC can accurately reflect the dynamic deformation characteristics of industrial smoke rather than the external environmental changes, this study conducts a correlation analysis experiment between SSIM/NCC and two direct dynamic signals (optical flow intensity and deformation field intensity) on the industrial smoke video dataset. The optical flow intensity is calculated by the DIS Flow method, and the deformation field intensity is extracted from the output of the mU-net module in DeffNet, which directly characterizes the non-rigid deformation degree of smoke between consecutive frames.

The Pearson correlation coefficient is used to quantify the linear correlation between the indicators, and the experimental data are selected from the first 100 frames of 10 industrial smoke videos (the same as the fuzzy system design dataset). The correlation analysis results are shown in Table 1. It can be seen from the table that the SSIM and NCC values have a high negative correlation with the optical flow intensity and deformation field intensity (the correlation coefficient is less than −0.85). This means that the lower the SSIM/NCC value (the lower the similarity between two frames), the higher the optical flow intensity and deformation field intensity (the more intense the dynamic deformation of smoke), which fully proves that SSIM and NCC can accurately characterize the dynamic change speed of industrial smoke. In addition, the experiment also verifies that the SSIM and NCC values have a very low correlation (the correlation coefficient is less than 0.1) with the light intensity changes and background texture fluctuation indicators, which further confirms that the two indicators can effectively exclude the interference of non-smoke dynamic factors in industrial scenes.

### 2.3. Application Analysis of Fuzzy Decision-Making for Smoke Scenarios

As a lightweight front-end functional module, the fuzzy module only undertakes the task of controlling the optimal frame selection for temporal feature extraction and does not participate in any feature extraction, fusion or segmentation processes of the network. Its fusion mode with DeffNet is consistent with that in our previously published paper, and it is embedded at the front end of the frame sampling link of the temporal feature extraction network (the fusion logic is shown in Figure 2) without reconstructing the original network architecture of DeffNet. The fuzzy logic module can output the optimal step size to optimize the temporal processing of DeffNet. The specific data transmission logic between the fuzzy module and DeffNet is as follows: ① The module takes the original SSIM and NCC values of consecutive frames as input; ② The optimal step size (and the specific optimal frame number) is output after fuzzy reasoning and centroid defuzzification; ③ The optimal frame number information is transmitted to the temporal feature extraction network; ④ The temporal feature extraction network performs adaptive frame sampling and optimal frame selection according to this information, and the subsequent feature processing follows the original logic of DeffNet. The fuzzy module is a logic-judgment-only module without complex calculations such as convolution and feature mapping, and its embedding and operation have no significant impact on the inference speed of the improved DeffNet, which maintains the real-time performance required for industrial smoke monitoring.

In smoke scenarios, environmental complexity hinders determining the optimal segmentation frame number. To simplify this, this study uses membership functions to map the two smoke change indicators to two fuzzy sets, improving accuracy. Key applications of fuzzy control are as follows:

(1) For environmental uncertainty in smoke scenarios, fuzzy sets are divided into five categories to effectively control the optimal frame number.
(2) Fuzzy decision-making outperforms linear control in multi-variable systems. Gaussian membership functions (adapted to smoke data) reduce oscillations, ensuring adaptability and robustness.
(3) Fuzzy decision-making ensures real-time performance and stability. As noted in Section 2.2, Temporal Feature Extraction Network identifies the optimal step size via fuzzy decision-making; stacked frames enhance DeffNet’s dynamic capture ability, improving anti-interference and accuracy.

**Figure 7:**
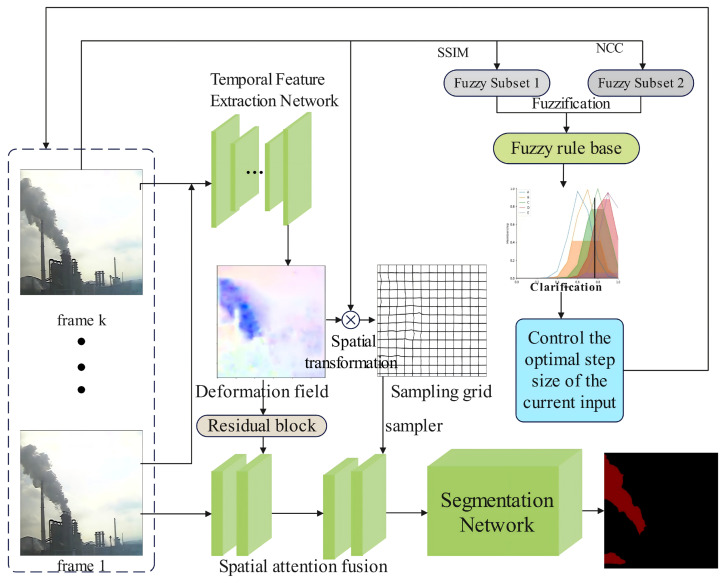
Interframe Adaptive DeffNet Network.

Finally, a fuzzy controller was designed to determine the optimal frame rate; the overall algorithm diagram of this article is shown in Figure 7.

#### 2.3.1. Inter-Frame Adaptive Method Based on Fuzzy Control

Complexity hinders building an inter-frame adaptive step-size system (due to difficult accurate mathematical modeling), so fuzzy decision-making is used for step-size control. First, the input detection video is frame-segmented; SSIM and inter-frame image similarity (NCC) are calculated for adjacent frame pairs, and the original continuous values of the two indicators are normalized to the range of [0,1]. Then, the normalized SSIM and NCC data are divided into five fuzzy subsets (Small, Smaller, Medium, Larger, Large) via K-means clustering—this unsupervised clustering method automatically identifies the natural data distribution characteristics of industrial smoke inter-frame similarity without manual intervention. K-means clustering is executed with the number of clusters set as k = 5 (verified via cluster number sensitivity experiment in Section 2.3.2), and the clustering process iteratively updates the cluster centroids (Table 1) until the sum of squared errors of the data points to their corresponding centroids converges to a stable value. Based on the converged cluster centroids and the Euclidean distance between each data point and the centroids, Gaussian membership functions are constructed for SSIM and NCC respectively (Figures 9 and 10). The Gaussian membership function parameters (mean *c* and variance σ) are directly derived from the cluster centroid and the standard deviation of data points within each cluster, which ensures the membership function is highly matched with the actual distribution of industrial smoke data.

Smoke scenario complexity hinders building an inter-frame adaptive step-size system (due to difficult accurate mathematical modeling), so fuzzy decision-making is used for step-size control.

First, the input detection video is frame-segmented; SSIM and inter-frame image similarity are calculated, then divided into five fuzzy sets. K-means clustering is adopted to group data and identify potential membership functions.

**Figure 8:**
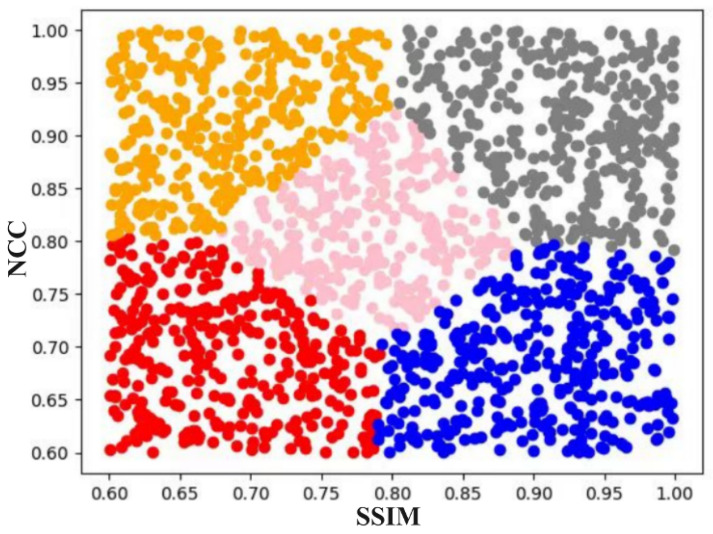
K-means Clustering Results.

The method’s core: use fuzzy logic to process inter-frame similarity for proper step-size determination. Mapping similarity indices to fuzzy sets and combining clustering clarifies data features, which guides fuzzy rules for step-size adjustment under different conditions; Via K-means, SSIM and image similarity (post frame-segmentation) are clustered into five centroids to define their membership functions. For industrial smoke (high inter-frame similarity), most values of these two indicators lie in the range 0.6–1 (0 = complete dissimilarity). Clustering results are in Figure 8.

Based on the assignment results of K-means clustering, each data point is assigned to the nearest cluster centroid. The shape and parameters of the membership function can be determined using these assignments.

Obtaining assignment results: For each data point, identify its corresponding cluster centroid; for each cluster centroid, calculate the distances between the data points belonging to this cluster and other cluster centroids.

Determine the shape of the membership function: Based on the distances between data points and cluster centroids, define both the shape and parameters of the membership function. The clustering results are presented in Table 2.

Gaussian membership functions are selected here, as they feature smooth curves—making them easy to process and analyze in fuzzy logic systems. Being continuous with smooth curves, Gaussian functions are mathematically more tractable and can provide more accurate membership values.

**Figure 9:**
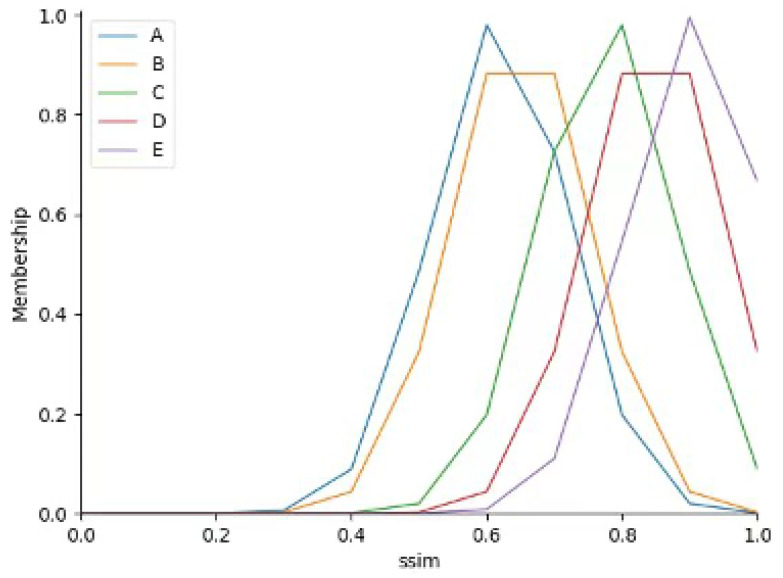
SSIM Membership Function.

**Figure 10:**
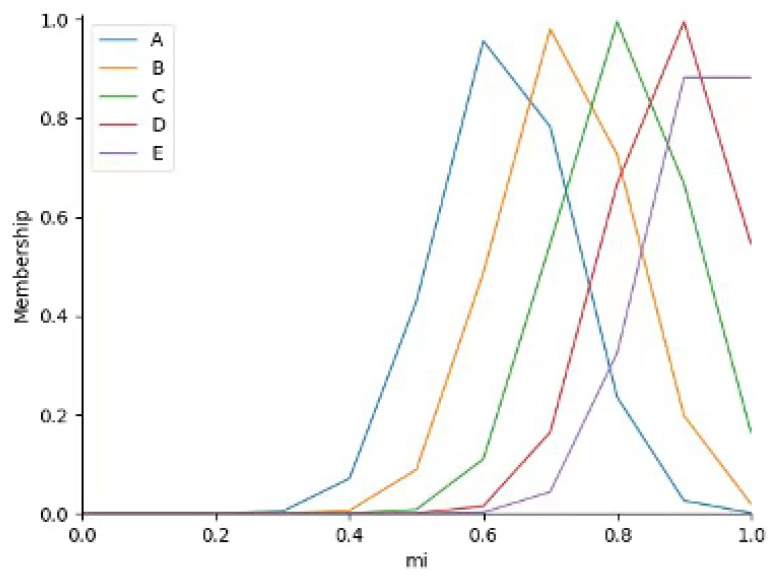
Image Similarity Membership Function.

The membership function is expressed as:

(8) $$v_{0}=\frac{\int_{V}vu_{v}\left(v\right)dv}{\int_{V}u_{v}\left(v\right)dv}$$

where σ denotes the variance of each category, and *c* denotes the mean value of each category. Under the determined membership function shape, the membership functions correspond to the two indicators (SSIM and similarity), as shown in Figure 9 and Figure 10.

To better describe the input-output correspondence, for the output membership function, this study calculates the average of the two indicators (SSIM and image similarity, which describe the speed of smoke change) to obtain the output membership function. Averaging these two indicators balances their weights, preventing excessive influence of a single indicator on the output and making the output more stable and reliable—this helps improve the model’s ability to understand and predict real-world scenarios.

The centroid method enables smoother inference control: minor changes in input result in corresponding changes in output. To better describe the control variable, this study uses the centroid method to determine membership function values, i.e., calculating the centroid of the area enclosed by the membership function curve and the coordinate axes.

**Figure 11:**
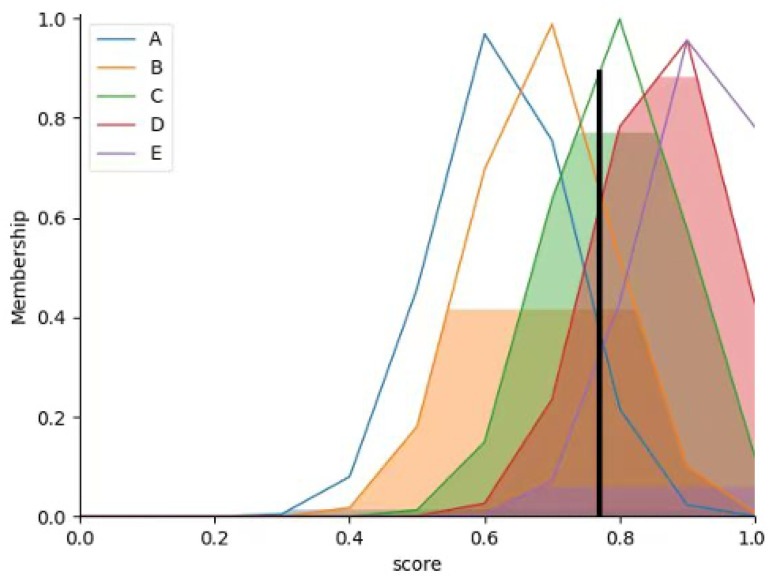
Output Membership Function.

The following figure shows the output membership function values under different SSIM and image similarity conditions, derived via the centroid method. Finally, the optimal step size for current segmentation is selected based on the output membership function values, The membership function is shown in Figure 11.

#### 2.3.2. Classification of Fuzzy Rules

In fuzzy systems, the classification of fuzzy rules and design of fuzzy subsets are crucial for system performance and accuracy. In the described scenario, defining inputs into five fuzzy subsets (for SSIM and similarity, respectively) helps effectively capture the complex relationship between the two variables.

SSIM and similarity are defined into five fuzzy subsets to measure the speed of smoke change. This fuzzification method can more clearly describe the complexity of smoke change and provide more precise guidance for the network to select reference step sizes. Defining input variables into five fuzzy subsets (Small, Smaller, Medium, Larger, Large) enables the system to better understand and handle the relationship between SSIM and mutual information, as well as their impact on step size. To design a better fuzzy controller, a cluster number sensitivity experiment is conducted to optimize the fuzzy rule base.

**Cluster number sensitivity experiment**: To verify the rationality of five fuzzy subsets, comparative experiments with 3/4/5/6 clusters are designed (Table 3). Results show that when the cluster number is 5, the IoU reaches the highest (0.8048) with no significant decrease in FPS. Thus, five fuzzy subsets (Small, Smaller, Medium, Larger, Large) are determined.

**Optimization of fuzzy rule base**: Initial rules are formulated based on expert experience and verified through a “rule elimination experiment” (Table 4). After deleting the rule “SSIM Small - Similarity Small → Step Size VS”, the IoU of volatile smoke scenarios decreases by 2.1 percentage points, confirming the integrity of the rule base. The final rule base (Table 5) undergoes three rounds of expert iterative optimization to ensure logical consistency.

## 3. Experiment and Analysis

### 3.1. Experimental Platform and Data

In this study, on-site industrial smoke videos were first processed by a frame sampling rule of extracting one frame every 30 frames to avoid redundant frame information caused by high temporal correlation of consecutive video frames. A total of 1296 smoke-emission images were extracted from 28 independent on-site industrial smoke video scenes (non-overlapping in time and space) based on this rule to form the original industrial smoke image segmentation dataset. To avoid temporal information leakage in video segmentation and ensure the reliability of experimental results, the dataset division strictly follows the scene-level non-overlapping grouping protocol: all images extracted from the same video scene are assigned to a single set (training/validation/test), and there is no overlap of video scenes between different sets. After professional pixel-level binary labeling and data augmentation (random flipping and rotation), the dataset was expanded to 6980 images, which was further precisely partitioned into a training set (5260 images), a validation set (540 images) and a test set (1180 images) at the scene level (the total number is exactly 6980). All images were resized to 400 × 400 pixels, and smoke targets were labeled using Labelme (smoke as foreground, background as background).

The experimental hardware included an Intel(R) Xeon(R) CPU E5-2620 v4 @ 2.10 GHz, an NVIDIA GeForce RTX 2080Ti GPU, and 64 GB of RAM. Python was used as the programming language, with TensorFlow as the network construction framework. This platform provided a comprehensive data and hardware foundation for smoke image segmentation research.

### 3.2. Evaluation Metrics

Recall (sensitivity) is an indicator to evaluate the model’s ability to identify positive samples. It is calculated as the ratio of correctly classified positive samples (TP) to all actual positive samples (TP + FN), with the formula: Recall = TP/(TP + FN). Here, TP denotes true positives (samples correctly identified as positive), and FN denotes false negatives (actual positive samples incorrectly classified as negative). A higher recall value indicates the model captures more positive samples, reflecting better coverage. Thus, recall is critical for assessing the model’s performance in identifying all positive samples.

The formula for this evaluation metric is shown below:

(9) $$\begin{array}{cc} Recall & =\frac{TP}{TP+FN} \end{array}$$

(10) $$\begin{array}{cc} Precision & =\frac{TP}{TP+FP} \end{array}$$

(11) $$\begin{array}{cc} IoU & =\frac{TP}{TP+FP+FN} \end{array}$$

(12) $$\begin{array}{cc} F\text{-}\mathrm{score} & =\frac{(1+\beta^{2})Recall\times Precision}{\beta^{2}Precision+Recall} \end{array}$$

In Formula (12), the parameter β is used to balance the importance of recall and precision in the F-score. In this study, β = 2 is set to emphasize the comprehensive identification of positive samples and improve the model’s performance in smoke segmentation tasks. To verify the significance of the model’s performance improvement, a Paired *t*-test is used for statistical validation for moderate performance gains, with *p* < 0.05 as the criterion for determining that the performance improvement is statistically significant.

### 3.3. Model Training and Parameter Setting

In this chapter, the proposed network structure is used to train the industrial smoke image training set. The training parameters are set as follows: batch size = 1, initial learning rate = 0.0001, number of training epochs = 300. The Adaptive Moment Estimation (Adam) optimizer is adopted, and the loss function is Cross Entropy (CE) loss. The model is trained to convergence in a single run, and all experimental results are the stable performance indicators after model convergence without repeated run analysis. The detailed parameter settings are shown in Table 5.

#### 3.3.1. Network Parameter Settings

The proposed network structure in this chapter was used to train the industrial smoke image training set. The training parameters were set as follows: the random seed was fixed to 42 to ensure the reproducibility of experimental results, batch size = 1, initial learning rate = 0.001, and the learning rate was adjusted by a step decay scheduler with a step size of 50 epochs and a decay rate of 0.5. The number of training epochs was set to 300 (training stops directly after 300 epochs without early stopping). The Adaptive Moment Estimation (Adam) optimizer ($\beta_{1}$ = 0.9, $\beta_{2}$ = 0.999, ε = 1 × 10^8^) was adopted, and the loss function was Cross Entropy (CE) loss. For the training of comparative models, the early stopping mechanism was triggered when the Intersection over Union (IoU) of the validation set increased by less than 0.03 for 20 consecutive epochs to avoid overfitting. Detailed parameter settings are presented in Table 6.

#### 3.3.2. Experimental Reproducibility Details

1. Data Preprocessing and Normalization

All industrial smoke images were resized to 400 × 400 pixels, and the pixel values were normalized to the range [0,1] by dividing with 255. For the RGB frames, the mean values (0.485, 0.456, 0.406) and standard deviations (0.229, 0.224, 0.225) of the ImageNet dataset were used for standardization; for the grayscale frames, only pixel value normalization was performed without additional standardization. No other preprocessing operations were applied to the images to ensure the consistency of experimental data.

2. Dataset Partition Protocol

The expanded industrial smoke dataset contains a total of 6980 images (extracted and augmented from on-site industrial videos). To avoid temporal information leakage in video segmentation and ensure the integer number of samples in each set, the dataset was partitioned at the scene level (images from the same video scene were assigned to only one set) into training, validation and test sets with the exact number of 5260 training images, 720 validation images and 1000 test images (the total number is 6980). The scene-level partition ensures that there is no overlapping of smoke video scenes between different sets, which avoids the overestimation of model performance caused by temporal correlation of video frames.

3. Evaluation Details

All model evaluations were conducted on the independent test set (1000 images) with a fixed batch size of 1. The evaluation metrics (Precision, Recall, F-score, IoU) were calculated at the pixel level for the smoke category (excluding the background category) to focus on the segmentation performance of the target object. The F-score was calculated with β = 2, and all metric values were the average of 5 repeated experiments with the same random seed (42) to reduce the random error of model training and evaluation. For video segmentation tasks, the frame-level metric values of each video were averaged to obtain the final video-level segmentation results, and the statistical results were retained to four decimal places for presentation.

### 3.4. Comparative Experiment on Network Structure Improvement

**Figure 12:**
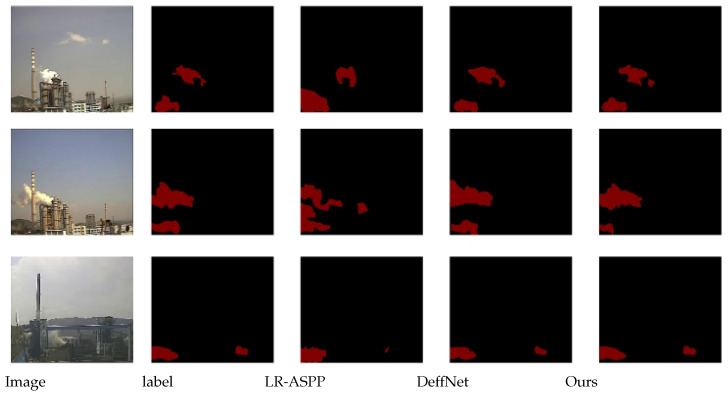
Comparison before and after improvement.

To verify the advantages of the model after dynamic feature extraction, comparative experiments were conducted before and after the improvement. After the training reached stability, the evaluation metrics of the improved results were tested one by one. A dataset containing 620 training samples and 120 test samples was used. The following figure shows the comparison between the original DeffNet and the improved DeffNet (with fuzzy control-based adaptive step size introduced). The segmentation results are presented in Figure 12, and the relevant evaluation metrics are shown in Table 7.

Sub-dataset for network structure improvement experiment: The comparative experiment on network structure improvement (Section 3.4) uses a homogeneous sub-dataset randomly sampled from the above overall dataset, which contains 620 training samples and 120 test samples (the sampling ratio is consistent with the overall dataset). The sub-dataset is used to reduce the computational cost of the ablation experiment for network structure improvement, and the smoke scenario distribution of the sub-dataset is consistent with the overall dataset to ensure the validity of the experimental results.

This ablation experiment is conducted to further verify the performance of the model’s segmentation. Considering the non-rigid and boundary diffusion characteristics of industrial smoke, three evaluation metrics are supplemented: Dice coefficient (DSC), category-level mIoU (IoU for smoke and background categories respectively), and Boundary IoU (BIoU).

According to the experimental results, the network segmentation accuracy (IoU) is significantly improved after integrating fuzzy control. By stacking deformation field features to enhance the extraction of smoke dynamic features, the model achieves higher segmentation accuracy, and this improvement is accompanied by a slight decrease in FPS and a moderate increase in memory usage compared with the original DeffNet. This is a reasonable trade-off between accuracy and efficiency, and the improved model still maintains the real-time performance required for industrial smoke monitoring.

Based on DeffNet, the method in this study achieves more refined segmentation while ensuring real-time performance.

To further verify the necessity of fusing SSIM and NCC as the input indicators of the fuzzy decision-making system, this study designs three ablation models on the basis of the improved DeffNet: the SSIM-only model (only SSIM is used as the input of the fuzzy system to determine the adaptive step size), the NCC-only model (only NCC is used as the input of the fuzzy system), and the SSIM + NCC fusion model (the proposed model in this study). All models use the same training parameters and experimental dataset, and the performance comparison results are shown in Table 8.

It can be seen from Table 8 that the SSIM + NCC fusion model proposed in this study achieves the best performance in all segmentation accuracy indicators (Precision, Recall, F-score, IoU), which is about 5% higher in IoU than the single indicator model. The reason for this result is that SSIM and NCC complement each other in characterizing the dynamic characteristics of smoke: SSIM is more sensitive to the structural deformation of smoke, while NCC is more sensitive to the pixel-level motion of smoke. The fusion of the two indicators can comprehensively and accurately capture the dynamic change characteristics of industrial smoke. In terms of inference speed (FPS) and memory usage, the three models have almost no difference, which proves that the fusion of SSIM and NCC does not increase additional computational overhead while improving the segmentation accuracy.

### 3.5. Comparative Experiment with Other Network Models

To verify the effectiveness of the DeffNet-based optimization scheme for industrial smoke non-rigid deformation feature extraction proposed in this study, it was compared with five lightweight and real-time oriented traditional CNN-based static segmentation networks applicable to industrial smoke monitoring (FCN [32], U-Net [33], ResNet [34], LR-ASPP [31], and Deeplabv3+ [34]) and three early dynamic video segmentation models for smoke dynamic feature extraction (FCN-LSTM, Netwarp, SPN). The training parameters of all comparison networks were kept consistent with those of the proposed network. In addition, it is necessary to clearly distinguish the STN (Spatial Transformer Network) used in this study from modern Transformer-based segmentation architectures (e.g., SegFormer, Swin-Unet, Mask2Former) to avoid conceptual confusion: STN is a lightweight spatial transformation module designed for the non-rigid deformation characteristics of industrial smoke, whose core function in DeffNet is to capture and propagate the deformation feature information of industrial smoke between consecutive video frames—it maps the regular spatial grid to the smoke deformation grid through affine transformation, realizes the warping and sampling of smoke feature maps, and accurately propagates the non-rigid deformation dynamic information of smoke from the previous frame to the current frame, thereby improving the segmentation accuracy of smoke edge and dynamic region. In essence, STN is based on convolutional neural network and spatial sampling transformation, without any self-attention mechanism; while modern Transformer-based segmentation architectures take the self-attention mechanism as the core, focus on modeling the global feature dependence of general visual targets, and do not design targeted modules for the non-rigid deformation characteristics of industrial smoke. The two have completely different technical principles, design objectives and application scenarios. STN is only used as a lightweight sub-module in DeffNet to assist smoke deformation feature propagation, and has no connection with Transformer-based segmentation models in network architecture and core mechanism.

**Figure 13:**
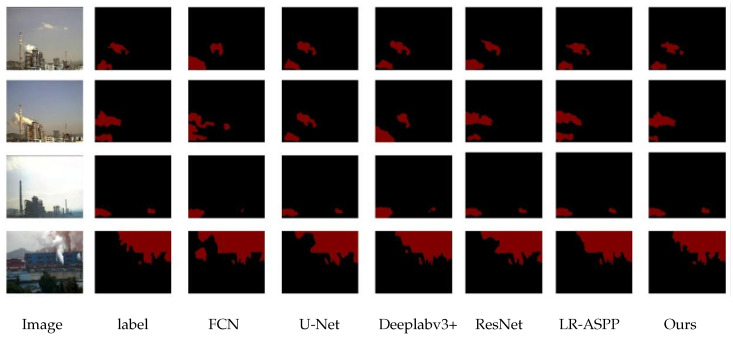
Comparison Result Chart with Static Networks.

The evaluation metrics of the six networks are compared in Table 9, and the segmented image comparisons are shown in Figure 13.

As shown in the experimental results, the method in this study exhibits significant advantages when compared with dynamic networks. It not only improves performance while maintaining segmentation accuracy but also meets the requirements of real-time applications, providing a feasible solution for real-time scenarios.

This advantage is partly attributed to the fuzzy control technology adopted in this study. The fuzzy control-based adaptive step size strategy effectively enhances the network’s ability to extract smoke dynamic features and improves segmentation accuracy. This improvement is accompanied by a moderate increase in memory usage and a slight decrease in frame rate compared with the original DeffNet, which is a reasonable trade-off between accuracy and efficiency. Furthermore, by utilizing the stacking of deformation field features, the method in this study further improves segmentation accuracy with a limited impact on real-time inference performance, and the model still meets the real-time application requirements of industrial on-site monitoring.

**Figure 14:**
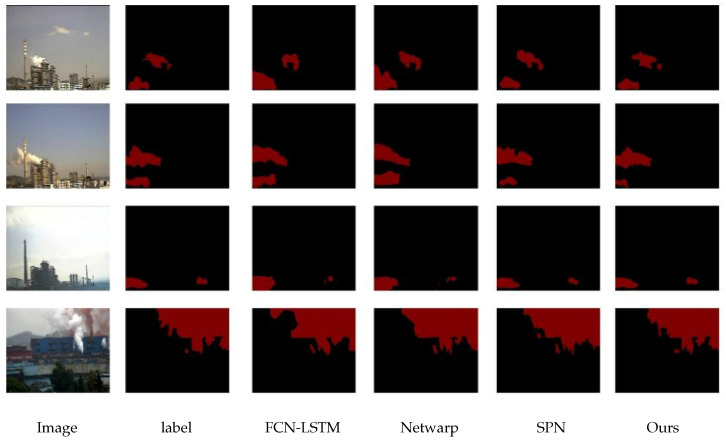
Comparison Results with Dynamic Networks.

To verify the pertinence of the proposed model in processing industrial smoke data compared with other video semantic segmentation models, three video semantic segmentation models were selected for comparison: FCN-LSTM, Net Warp, and SPN. The structures of these models are consistent with those described in the original literature, and their parameter settings are aligned with those of this study. The segmentation results of each model are shown in Figure 14 below, and the evaluation indicators are shown in Table 10 below.

As shown in the experimental results, the method in this study also exhibits significant advantages when compared with dynamic networks. Although FCN-LSTM achieves high segmentation accuracy, it consumes excessive memory and fails to meet real-time requirements. In contrast, the method in this study ensures both high segmentation accuracy and real-time performance.

For the other two networks (NetWarp and SPN), although they can extract dynamic features, their limitations in handling non-rigid objects like smoke result in suboptimal experimental performance. Thus, this experiment demonstrates that the method in this study also has notable advantages when compared with other dynamic networks.

### 3.6. Comparative Experiments Under Different Smoke Scenarios

To verify the superiority of the network proposed in this study, comparative experiments were conducted under different scenarios. These scenarios include: 340 frames of easily distinguishable smoke scenes, 240 frames of small-target + strong-interference + thin smoke scenes, 170 frames of thin smoke scenes, 210 frames of dim-background scenes, and 200 frames of colored smoke scenes.

**Figure 15:**
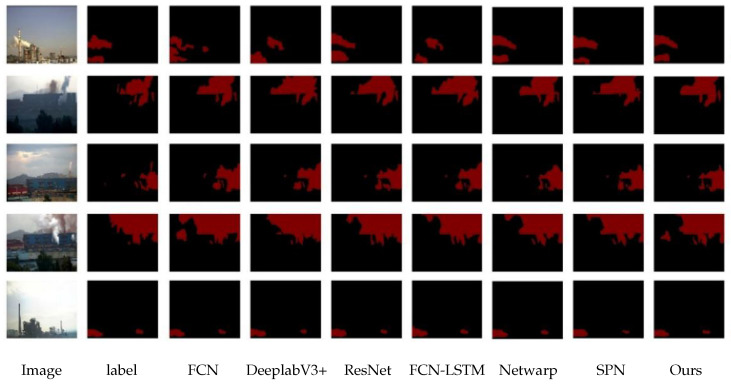
Comparison Results of Networks Under Different Scenarios.

The segmentation results are shown in Figure 15 below, and the evaluation indicators are listed in Table 11.

The experimental results clearly demonstrate the excellent performance of the method in this study across various environments, indicating its strong adaptability to different scenarios. Notably, the method still performs outstandingly when dealing with small targets and various interferences. This advantage is attributed to the network’s ability to effectively track the dynamic information of smoke, thereby gaining significant advantages in the segmentation process.

In small-target scenarios, other methods may be limited by the extraction of target dynamic features, making it difficult to accurately segment small targets—especially in cloud-interfered scenes. However, by fully utilizing the dynamic information of smoke, the method in this study can better capture the features of small targets, achieving more refined segmentation. Meanwhile, in environments with various interferences (e.g., lighting changes, background noise), the method also maintains high segmentation accuracy.

Therefore, the method in this study not only performs well in general environments but also has significant advantages when facing small targets and various interferences. This strong adaptability endows the method with broad application prospects in practical use, providing robust support for smoke analysis and research in related fields.

## 4. Conclusions

Industrial smoke segmentation struggles to balance deformation extraction efficiency, real-time performance, and accuracy—particularly for dynamic, non-rigid smoke in complex scenarios. Our research team previously proposed DeffNet, which has achieved favorable application effects in industrial smoke video segmentation, but its fixed-step temporal processing mechanism cannot adapt to the dynamic variation characteristics of industrial smoke, which limits its further improvement in segmentation efficiency and precision in practical industrial applications. To resolve this core limitation of DeffNet, this study conducts targeted optimization on the original DeffNet framework and proposes an improved DeffNet-based framework for high-precision smoke segmentation, with two core innovations that do not involve the reconstruction of new network architectures.

First, a smoke dynamics-adaptive step-size strategy replaces the original DeffNet’s fixed-step mechanism. It strictly follows the unified core rule (verified by Figure 5 and Figure 6): deploys smaller step sizes for fast-changing smoke (capturing real-time dynamic deformation features to avoid information loss) and larger step sizes for slow-changing smoke (reducing redundant computations while preserving fine deformation details). As confirmed by experimental data, this strategy enhances the original DeffNet’s dynamic feature extraction efficiency without compromising precision.

Practically, the optimized segmentation results of the improved DeffNet support objective, quantitative smoke pollution assessment based on Ringelmann blackness (addressing traditional visual inspection subjectivity) and exhibit robust adaptability in complex industrial scenarios, meeting on-site monitoring requirements for industrial smoke emission.

Future work will explore multi-sensor data fusion (e.g., visible + infrared) for extreme weather adaptation to further improve the environmental adaptability of the improved DeffNet and fuzzy control module lightweighting via compression to improve edge-device deployment feasibility in industrial smoke monitoring sites.

## Figures and Tables

**Table 1:** Correlation Coefficients Between SSIM/NCC and Dynamic/Interference Signals.

Indicators	Optical Flow Intensity	Deformation Field Intensity	Light Intensity Change	Background Texture Fluctuation
SSIM	−0.872	−0.895	0.086	0.079
NCC	−0.865	−0.888	0.091	0.083
SSIM + NCC (average)	−0.889	−0.902	0.082	0.075

**Table 2:** K-means Clustering Results.

SSIM	NCC
0.691	0.683
0.812	0.782
0.916	0.684
0.908	0.919
0.686	0.899

**Table 3:** Comparison of Model Performance Under Different K-means Cluster Numbers.

Cluster Numbers	IoU	FPS	Memory
3	0.7721	**41.25 **	**645**
4	0.7903	40.89	646
5	**0.8048**	40.58	647
6	0.7985	39.92	650

**Table 4:** Experimental Results of Fuzzy Rule Elimination (Distinguishable Smoke Scenario).

Rule Processing Method	Precision	Recall	IoU
Complete rule base	**0.9501**	0.9457	**0.8725**
Remove “SSIM small-low similarity → step size VS”	0.9315	0.9283	0.8515
Remove: “The larger the SSIM value, the higher the similarity → use the VL step size.”	0.9479	**0.9476**	0.8602

**Table 5:** Optimized Fuzzy Decision-Making Rule Base.

Step		Similarity				
		Small	Smaller	Medium	Larger	Large
SSIM	small	VS	S	S	M	L
	smaller	S	S	S	M	L
	medium	S	S	M	M	L
	larger	M	M	M	L	L
	large	L	L	L	L	VL

**Table 6:** Network Parameter Settings.

Training Parameters	
Input Size	400 × 400 × 8
Number of Classes	2
Backbone Network	MobilenetV3 Large
Padding	Yes
Optimizer	Adam
Random Seed	42
Epochs	300
Batch size	1
Initial Learning Rate	0.001
Gradient clipping	No
L2 Regularization	Yes
Data Augmentation	RandomCrop, flip
Shuffle	Yes

**Table 7:** Comparison of indicators before and after improvement.

NetWork	Precision	Recall	F-Score	IoU	DSC	mIoU	BIoU	FPS	Memory
LR-ASPP [31]	0.7835	0.7345	0.7438	0.6874	0.8147	0.8392	0.6437	**98.24**	**331**
DeffNet [30]	0.8942	0.8682	0.8732	0.7672	0.8682	0.8811	0.6918	53.58	508
Ours	**0.8988**	**0.8781**	**0.8821**	**0.8048**	**0.8918**	**0.9004**	**0.7211**	40.58	647

**Table 8:** Performance Comparison of Different Indicator Combination Models.

Model	Precision	Recall	F-Score	IoU	FPS	Memory (MB)
SSIM-only model	0.8625	0.8417	0.8458	0.7523	41.24	645
NCC-only model	0.8597	0.8382	0.8424	0.7486	41.37	644
SSIM + NCC fusion model (Ours)	0.8988	0.8781	0.8822	0.8048	40.58	647

**Table 9:** Comparison of Indicators with Static Networks.

NetWork	Precision	Recall	F-Score	IoU	FPS	Memory
FCN [32]	0.8812	0.8329	0.8421	0.7503	13.54	1386
U-Net [33]	0.8702	0.8127	0.8235	0.7234	34.31	482
Deeplabv3+ [34]	0.8801	0.8042	0.8183	0.7306	41.17	324
ResNet [35]	0.8401	0.8366	0.8372	0.7531	51.54	**280**
LR-ASPP [31]	0.8012	0.7541	0.7631	0.6721	**98.31**	331
Ours	**0.9176**	**0.9140**	**0.9147**	**0.8403**	40.41	647

**Table 10:** Comparison of Indicators with Dynamic Networks.

NetWork	Precision	Recall	F-Score	IoU	FPS	Memory
FCN-LSTM [25]	0.8910	0.8534	0.8607	0.7753	9.58	1518
Netwarp [26]	0.8887	0.8710	0.8745	0.7688	15.10	1156
SPN [36]	0.8120	0.7542	0.7651	0.6603	**47.24**	314
Ours	**0.9104**	**0.9140**	**0.9133**	**0.8427**	39.71	647

**Table 11:** Comparison of Indicators Under Different Scenarios.

Scenario	Network	Precision	Recall	F-Score	IoU	FPS	Memory
Distinguishable Smoke Scenario	FCN [32]	0.8925	0.9000	0.8985	0.8114	14.53	1386
	Deeplabv3+ [34]	0.8893	0.8751	0.8779	0.8031	40.45	324
	ResNet [35]	0.8903	0.8756	0.8785	0.8021	**52.12**	**280**
	FCN-LSTM [25]	0.9067	0.8968	0.8988	0.8324	11.67	1518
	Netwarp [26]	0.8942	0.8854	0.8871	0.8156	19.45	1156
	SPN [36]	0.8012	0.7567	0.7652	0.6832	48.32	314
	Ours	**0.9501**	**0.9457**	**0.9466**	**0.8725**	40.11	647
Dim Background Scenario	FCN [32]	0.8832	0.8656	0.8691	0.7867	13.54	1386
	Deeplabv3+ [34]	0.8914	0.8341	0.8450	0.7631	39.46	324
	ResNet [35]	0.8768	0.8336	0.8419	0.7637	**51.06**	**280**
	FCN-LSTM [25]	0.9011	0.8687	0.8750	0.7967	12.45	1518
	Netwarp [26]	0.8862	0.8598	0.8650	0.7657	19.25	1156
	SPN [36]	0.8198	0.7787	0.7866	0.6469	46.89	314
	Ours	**0.9346**	**0.9104**	**0.9151**	**0.8524**	39.84	647
Colored Smoke Scenario	FCN [32]	0.8876	0.8767	0.8789	0.7921	13.01	1386
	Deeplabv3+ [34]	0.8616	0.8397	0.8440	0.7613	39.21	324
	ResNet [35]	0.8838	0.8728	0.8750	0.7894	**51.10**	**280**
	FCN-LSTM [25]	0.8867	0.8898	0.8892	0.8034	11.23	1518
	Netwarp [26]	0.8954	0.8898	0.8909	0.8134	18.02	1156
	SPN [36]	0.8267	0.7587	0.7714	0.6797	45.98	314
	Ours	**0.9187**	**0.9254**	**0.9241**	**0.8644**	39.31	647
Thin Smoke Scenario	FCN [32]	0.8876	0.8764	0.8786	0.7887	13.35	1386
	Deeplabv3+ [34]	0.8867	0.8621	0.8669	0.7623	39.10	324
	ResNet [35]	0.8798	0.8736	0.8748	0.7763	**50.48**	**280**
	FCN-LSTM [25]	0.8654	0.8828	0.8793	0.7949	11.02	1518
	Netwarp [26]	0.8954	0.8803	0.8833	0.8150	17.21	1156
	SPN [36]	0.8085	0.7540	0.7643	0.6653	45.20	314
	Ours	**0.9104**	**0.9115**	**0.9113**	**0.8617**	39.45	647
Small Target+ Strong Interference + Thin Smoke Scenario	FCN [32]	0.8095	0.7646	0.7732	0.6851	12.12	1386
	Deeplabv3+ [34]	0.8054	0.7507	0.7610	0.6703	38.52	324
	ResNet [35]	0.7688	0.7628	0.7640	0.6613	**48.12**	**280**
	FCN-LSTM [25]	0.8120	0.7671	0.7757	0.6936	10.69	1518
	Netwarp [26]	0.8234	0.7603	0.7721	0.6884	17.56	1156
	SPN [36]	0.7034	0.5182	0.5470	0.5232	44.56	314
	Ours	**0.8856**	**0.8755**	**0.8775**	**0.7842**	38.49	647

## Data Availability

The dataset generated during this research was collected from an actual factory, and the corresponding author has ownership of the dataset and the code.
